# Curiouser and curiouser: understanding the intersection of inter- and intra-specific diversity during the *Borrelia burgdorferi* enzootic cycle and Lyme disease

**DOI:** 10.1128/iai.00554-25

**Published:** 2026-08-04

**Authors:** Marina S. Winter, Kareem D. Adams, Jeffrey S. Bourgeois

**Affiliations:** 1 Department of Biology and Biotechnology, Worcester Polytechnic Institute 8718, Worcester, Massachusetts, USA; 2 Bioinformatics and Computational Biology Program, Worcester Polytechnic Institute 8718, Worcester, Massachusetts, USA; University of Pittsburgh, Pittsburgh, Pennsylvania, USA

**Keywords:** *Borrelia*, *Borreliella*, human genetics, diversity, reservoir, tick, Lyme disease

## Abstract

A consequence of the *Borrelia* (*Borreliella*) *burgdorferi* lifecycle is that a “standard” host-pathogen interaction involving the spirochete is challenging to define. The bacteria continuously cycle between vertebrate and invertebrate hosts, each inflicting substantially different selective pressures on the spirochete. Focusing solely on vertebrate infections, the spirochete can be found in numerous hosts in nature, including deermice, shrews, birds, and lizards. The immune pressures that the bacteria face in each of these hosts are variable and are thought to drive broad intraspecific variation across *B. burgdorferi* strains—with different strains appearing to be avian adapted, mammalian adapted, or generalist. In addition to its natural zoonotic hosts, *B. burgdorferi* can infect humans and cause Lyme disease. Here, natural human genetic variation can drive dramatically different disease outcomes, as some individuals seem capable of killing the bacteria before it can successfully colonize, while others are predisposed to continue reacting to the pathogen even long after it is killed by antibiotics. In this review, we describe studies investigating how host and bacterial diversity affect *B. burgdorferi* infection outcomes, with an emphasis on how this variability impacts the bacteria’s spread in nature and Lyme disease severity during human infection.

## INTRODUCTION

**Fig 1:**
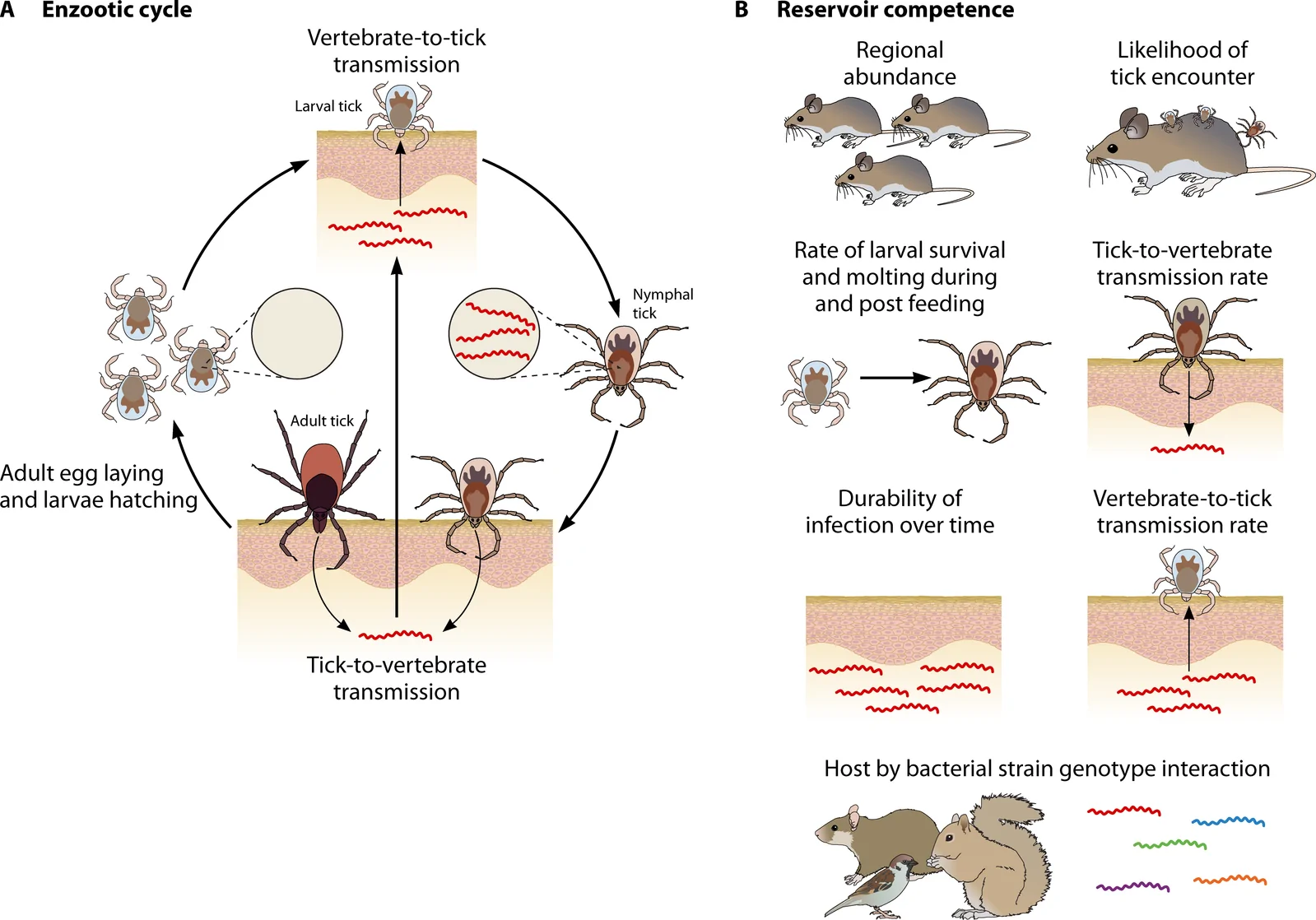
*B. burgdorferi* enzootic cycle and reservoir competence. (**A**) The *B. burgdorferi* enzootic cycle. Larval ticks (*Ixodes*) acquire *B. burgdorferi* during feeding on infected vertebrate hosts. These ticks molt to nymphs before feeding on uninfected hosts and transmitting the bacteria. During feeding, adult female ticks can also transmit the bacteria to reservoirs before completing the tick reproductive cycle and producing new, uninfected larval ticks. Reservoir hosts infected during nymphal and adult tick feeding can harbor the bacteria and, in turn, spread *B. burgdorferi* back into the tick population. (**B**) Reservoir competence depends on a variety of factors, including abundance of the host, host-tick interactions, and host-*B. burgdorferi* interactions (7). Additional complexity is added to the system as different hosts may be more or less competent for specific strains of *B. burgdorferi* (13, 14). Figure illustrated by Patrick Lane—ScEYEnce Studios.

The outcome of any host-pathogen interaction is the product of the genetic background of the host, the genetic background of the pathogen, environmental and life history factors, and random chance. Small genetic variations in either the host or pathogen can dramatically impact whether a host resists infection and/or develops active disease. Despite these variables, for many pathogens, we are able to develop useful frameworks of “canonical” host-pathogen interactions, which broadly describe how these interactions usually play out. By definition, this also enables us to identify “non-canonical” host-pathogen interactions, where variables can shift the interaction in favor of either the host or pathogen. However, these broad but convenient schematics begin to buckle and bend for pathogens with wide host ranges, as one needs to consider the impact of genetic variation between infected individuals belonging to a single host species, as well as the dramatically different genetic and physiological backgrounds of different host species. For Lyme disease-causing spirochetes, including *Borrelia* (*Borreliella*) *burgdorferi*, impacts of inter- and intra-specific host and microbe diversity are inherently tied to the bacteria’s lifecycle. *B. burgdorferi* propagates through an enzootic cycle: a continuous transmission chain whereby uncolonized *Ixodes* ticks feed on infected vertebrates, acquire the bacteria, and spread the bacteria back to uninfected vertebrates during future feedings (Fig. 1A). During this cycle, *B. burgdorferi* is almost an accidental tourist in nature, moving between hosts not by its own agency, but by the feeding choices of its tick vector. Critically, *Ixodes* ticks only feed three times in their lives, and there is no transovarian spread of *B. burgdorferi* (1), meaning the opportunities for *B. burgdorferi* to transmit from a tick host are limited. Furthermore, because *Ixodes* are generalist ectoparasites (2), *B. burgdorferi* must attempt to continue its enzootic cycle with whichever vertebrate host the tick feeds on—with varying degrees of success. Once in a vertebrate host, the bacteria must evade host immunity long enough (likely weeks or months, based on the feeding patterns of *Ixodes* ticks [3–5]) for the animal to be fed on by new larval or nymphal ticks. For extant strains, these interactions have played out over and over and over again as *B. burgdorferi* has continued through its enzootic cycle. At each step, these spirochetes leverage their small genetic toolkit to combat highly variable immune systems in avian, reptilian, or mammalian hosts (6–12). A particularly important interaction arises when ticks feed on human hosts, enabling zoonotic spillover into humans and the development of Lyme disease. Here too, intra-specific diversity across human hosts and bacterial strains has dramatic impacts on disease outcomes.

In this review, we discuss the impacts of host and bacterial diversity on *B. burgdorferi* transmission and pathogenesis. We summarize what is known and unknown about how *B. burgdorferi* biology shifts across different vertebrate hosts, note how different bacterial strains interact with wild and human hosts, and identify future directions in understanding *B. burgdorferi* enzootic cycling and Lyme disease pathogenesis.

## UNDERSTANDING *BORRELIACEAE* NATURAL DIVERSITY

*B. burgdorferi* is a member of the spirochete family *Borreliaceae* (15). Almost all pathogens within the family are tick borne, and these spirochetes cluster into three major lineages: relapsing fever-causing spirochetes, Lyme disease-causing spirochetes, and a genetically distinct lineage of reptile-associated *Borrelia* spirochetes (15, 16). Genomic work has demonstrated that these divergent clinical manifestations are the result of the pathogens being separate monophyletic groups (17–19), which has led some (15, 18, 19) to favor splitting *Borreliaceae* into at least two independent genera: *Borrelia* (the clade containing relapsing fever spirochetes and reptile-associated *Borrelia*) and *Borreliella* (the clade containing Lyme disease spirochetes). This nomenclature has been unevenly applied within the literature, with some arguing against this nomenclature (either directly [20, 21] or by omission) in favor of the original nomenclature. Traditionally, Lyme disease spirochetes are clustered in genospecies under *Borrelia burgdorferi* sensu lato—including the genospecies *Borrelia burgdorferi sensu stricto* (spirochetes related to the original *B. burgdorferi* strain isolated from a Long Island, New York tick [22, 23]), while relapsing fever spirochetes belong to different species. Under the *Borreliella* nomenclature, these *sensu lato* strains are classified as distinct species, avoiding confusion between *Borrelia burgdorferi sensu stricto* and genetically distinct Lyme disease spirochetes. A compromise solution has been proposed in which *Borreliella* is listed as a subgenus, rather than a new genus (24). In this work, which focuses only on Lyme disease spirochetes, we will refer to *Borrelia burgdorferi* sensu stricto*/Borreliella burgdorferi*/*Borrelia* (*Borreliella*) *burgdorferi* as *B. burgdorferi*, and *Borrelia burgdorferi* sensu lato/other *Borreliella* species by their *Borrelia* genospecies nomenclature (e.g., *Borrelia garinii*).

The prevalence of different Lyme disease spirochetes varies dramatically across continents, with *B. burgdorferi* being the primary (though not exclusive) genospecies in North America (25), while multiple genospecies are present in high abundance throughout Eurasia (e.g., *Borrelia afzelii*, *B. garinii*, *B. burgdorferi*, among others [26]). Recent advancements in genomics have dramatically improved our ability to compare both the diversity across these Lyme disease spirochetes as well as the diversity within a given genospecies. Comparing genospecies, recent work has identified a core Lyme disease spirochete genome, which includes much of its linear chromosome (17). Meanwhile, accessory components on the genome are carried on the large number of linear and circular plasmids housed by *Borreliaceae* spirochetes.

**Fig 2:**
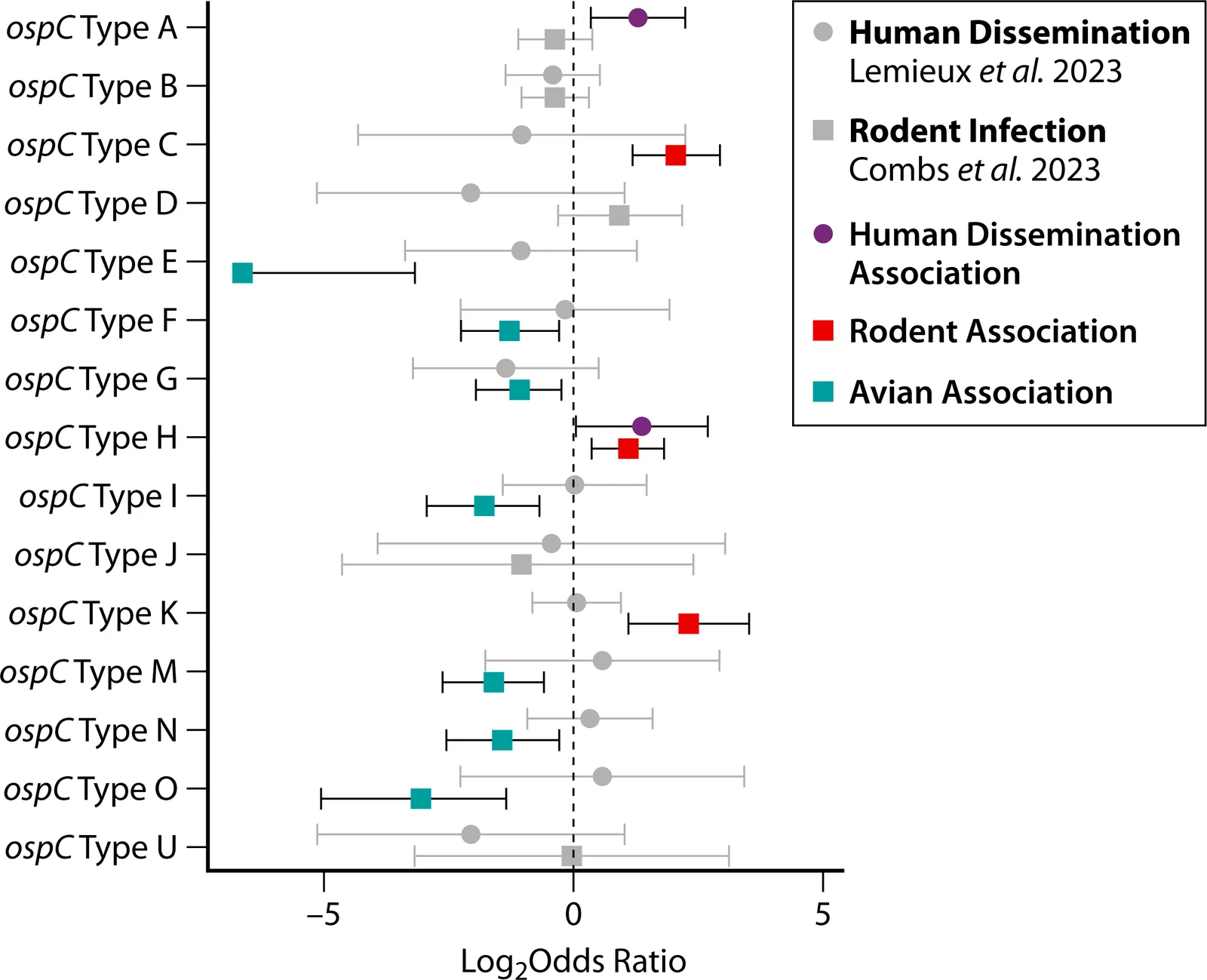
*B. burgdorferi* natural diversity impacts Lyme disease outcomes and enzootic cycling success. *ospC* genotypes partially predict human dissemination in patients (36) and prevalence in reservoir hosts (13). Circles represent human dissemination odds ratios (calculated based on S4 Table by Lemieux et al. [36]) and squares report odds ratios for rodent infection by Combs et al. (13). Violet circles represent an increased association with human dissemination, red squares denote increased association with rodent hosts, each determined by a positive statistically significant odds ratio (*P* < 0.05). Blue squares represent strains with higher association with avian hosts, determined by a negative statistically significant odds ratio (*P <* 0.05). Error bars represent the 95% CI. Only *ospC* genotypes with odds ratios in both studies are shown in this figure. Figure remastered by Patrick Lane—ScEYEnce Studios.

Within *B. burgdorferi* itself, intraspecific genetic diversity driving overall phenotypic diversity has long been appreciated. Early genetic studies successfully clustered *B. burgdorferi* strains into clinical presentations according to one of two single-loci genotypes: one of the approximately 30 genotypes at the lipoprotein *ospC* locus (*ospC* type) (27, 28) or one of the three ribosomal spacer region (RST) genotypes (29). *ospC* type A strains (a member of the RST1 subset) are highly pathogenic in humans and are associated with more severe disease as well as elevated risk of antibiotic-refractory disease (30), as we explore in greater detail below (Fig. 2). A multilocus sequence typing (MLST) system has also been described, examining eight chromosomal genes and grouping *B. burgdorferi* into over 900 sequence types (31, 32). A recent large-scale (299 isolates) genomic study by Lemieux et al., utilizing short-read DNA sequencing, demonstrated that while most (but not all, likely due to recombination [33–35] among distantly related strains) of these *ospC* types form monophyletic clades, among the RST genotypes, only RST1 is a monophyletic group (36). Using this whole-genome data, an additional (WGS) classification was proposed, separating *B. burgdorferi* strains into four monophyletic groups. Under this classification, WGS Group A contains the highly pathogenic *ospC* Type A/RST1 clade. While short-read genomic sequencing can report associations between the presence and absence of genes and phenotypes (36), the *B. burgdorferi* plasmids, which can feature high homology and recombination rates, cannot be easily distinguished using these techniques. Studies have overcome these difficulties by pairing short- and long-read sequencing (37–40). Key findings from these studies include limited variability on the *B. burgdorferi* chromosome, but dramatic variation in the specific plasmid profiles across isolates, with strong conservation of “gene blocks” or sets of genes that appear to be linked—often correlating with a specific plasmid (36, 37). Evolutionary genomics data suggest that lipoproteins on these plasmids, which make up approximately 7.8% of the genetic content of these plasmids (41), likely play a role in many cross-strain and cross-genospecies differences (17, 36, 37, 42).

Of note, the ϕBB-1 prophage in *B. burgdorferi*, encoded by the circular plasmid 32 family, is capable of facilitating homologous and heterologous DNA horizontal gene transfer (33, 43–45). These phage particles are produced during the tick stage of infection (46), which is notable as ticks can frequently be colonized by multiple Lyme disease spirochetes (13, 47–51). This offers ample opportunity for recombination events to provide selective advantages to strains preparing to reenter a vertebrate host and may be a key mechanism by which diverse combinations of plasmids and gene content are generated and maintained in *B. burgdorferi*.

## INTERACTIONS BETWEEN *B. BURGDORFERI* AND INTERSPECIFIC HOST DIVERSITY

### Studying diverse host-pathogen interactions in nature

The persistence of *B. burgdorferi* in nature is a consequence of chance and adaptation. Upon entry into a vertebrate host, *B. burgdorferi* faces unique pressure from the innate and adaptive immune systems of the particular host, while it attempts to survive long enough to complete another rotation of the enzootic cycle. Host-*B. burgdorferi* interactions during early infection effectively categorize vertebrate hosts as “competent” or “incompetent” reservoirs, based on whether they can both become infected with *B. burgdorferi* and transmit the bacteria to new ticks (Fig. 1B). Seminal work from LoGiudice et al. leveraged fieldwork and modeling to consider how the abundance of different species, the susceptibility of these species to *B. burgdorferi*, the capacity for those species to transmit *B. burgdorferi* to ticks, and the ability for hosts to support tick feeding ultimately drive human Lyme disease risk by impacting the number of ticks in a region capable of enabling zoonotic spillover (8). Species such as deermice (*Peromyscus* species, including *Peromyscus leucopus*) (11) and the short-tailed shrew (*Blarina brevicauda*) (12) exhibit very high reservoir competence, acting to amplify the amount of *B. burgdorferi* circulating in a region. On the other hand, hosts like the eastern gray squirrel (*Sciurus carolinensis*) show very low reservoir competence (8). White-tailed deer (*Odocoileus virginianus*) likewise can be fed on by various *Ixodes scapularis* tick stages (52, 53) but are incapable of serving as *B. burgdorferi* reservoirs (8, 53, 54). As such, these low-competence hosts “dilute” the abundance of *B. burgdorferi* in a region by essentially “wasting” a stage of tick feeding that could have resulted in *B. burgdorferi* reentering the enzootic cycle, reducing overall human disease risk in the area. Importantly, whether a host species exerts a meaningful dilution effect depends on two factors: its reservoir competence (or incompetence) and the burden of larval ticks it feeds. A highly incompetent host that rarely feeds ticks will have negligible impact on enzootic transmission, whereas a moderately competent host that feeds enormous numbers of ticks could still amplify disease risk. Thus, estimating dilution potential requires integrating field measurements of tick burden, host population density, and reservoir competence (Fig. 1B).

Reptiles offer another well-characterized example of dilution effects in Lyme disease ecology. On the west coast of the United States, the western fence lizard (*Sceloporus occidentalis*) is a common host for immature *Ixodes pacificus* ticks but is highly refractory to *Borrelia burgdorferi* infection due to a complement-mediated borreliacidal factor in its blood (55, 56). This reservoir incompetence, combined with the large numbers of larval ticks that feed on these lizards, allows western fence lizards to function as dilution hosts, reducing the prevalence of infected nymphs in habitats where they are abundant (57). Giery and Ostfeld (2007) demonstrated that the five-lined skink (*Eumeces fasciatus*) feeds substantial numbers of larval *Ixodes scapularis*, yet xenodiagnosis trials revealed that none of the 164 ticks that fed on wild-caught skinks acquired *B. burgdorferi* (58). Using an empirical model, Giery and Ostfeld (2007) calculated that the presence of skinks in a host community reduces nymphal infection prevalence by 10.7%–51.5% (58).

Substantial work examining reservoir competence, including much of the work discussed throughout this review, has examined the frequency of infected ticks found feeding on a specific host—typically by bringing trapped animals back to the laboratory where ticks can feed to repletion (8, 59). Recent studies have begun leveraging a PCR-based approach (60–62) to measure reservoir competency from non-host-associated ticks in the wild. This method targets vertebrate species-specific retrotransposon elements that persist in ticks following bloodmeal digestion, allowing researchers to infer which species the ticks fed on across multiple life stages. This allows analysis of ticks not actively feeding, detection of feeding on hosts that are difficult to wild-capture, and offers the ability to detect mixed bloodmeals from multiple host species fed upon by a single tick. Using this method, Goethert et al. tested ticks from Nantucket Island and Martha’s Vineyard for molecular evidence of which hosts the ticks had fed on and the presence or absence of *B. burgdorferi* DNA. Using this approach, they found that while ticks most frequently fed on *Peromyscus* and deer, albeit with substantial variation across years, shrews were critical for *B. burgdorferi* transmission. In their Nantucket study, shrews fed only 12% of larval ticks but were the source of 31% of infected nymphs (54). Notably, these data highlight the role of “minor” hosts in *B. burgdorferi* transmission, contrasting with the modeling by LoGiudice et al., which instead supported the long-held convention that *P. leucopus* serves as the dominant reservoir for *B. burgdorferi* (11, 63). We speculate that these differences are likely due to the improved sampling of shrew-fed ticks achieved by capturing post-feeding ticks. While additional work is necessary to confirm these findings, including testing the ticks that feed on these hosts for viable *B. burgdorferi*, they highlight that the enzootic cycle is robust to individual species changes and that the exact route *B. burgdorferi* takes through the cycle depends greatly on community composition.

A specific and immediate innate immune challenge that *B. burgdorferi* must overcome in potential reservoir hosts is complement-mediated killing. While the classical complement cascade can kill *B. burgdorferi* late in infection via the binding of adaptive immunity-generated antibodies, the alternative complement cascade has the capacity to kill *B. burgdorferi* from the first moments of infection (64). Consequently, complement killing appears to correlate with reservoir competence (65). For instance, serum from reservoir-incompetent white-tailed deer is rapidly and potently borreliacidal, killing 100% of spirochetes from multiple, genetically distinct strains *in vitro* within hours (66). This effect appears to be largely complement dependent, as heat-inactivation significantly reduces killing activity (66). This complement-mediated defense is conserved across cervids, as shown by similar borreliacidal activity in sika deer serum (67). In this latter work, the use of EDTA and EGTA chelation demonstrated that the alternative complement cascade, but not the classical complement cascade, is likely responsible for this killing (67).

While reservoir-incompetent hosts are incompetent for all strains of *B. burgdorferi*, competent reservoir hosts occupy highly variable positions. Work by Marcinkiewicz et al. demonstrated that variation in the *B. burgdorferi* complement evasion factor CspZ enabled survival in either mammalian or avian serum, as well as dissemination in either mouse or quail hosts (68). Though the ability for *B. burgdorferi* and other Lyme disease spirochetes to resist complement is predictive in some cases of when a spirochete can complete tick-to-vertebrate-to-tick transmission in a given host (66, 69–72), Lin et al. noted that susceptibility to *P. leucopus* serum did not predict transmission (72). Thus, other factors also play a role in determining host competence, and these additional barriers to enzootic cycling success remain an area of active investigation.

Whether complement driven or not, these observations fit into a broader narrative of genetic variation as a determinant in whether any individual strain of *B. burgdorferi* can use a given host as a reservoir. One explanation for these results is the “multiple niche polymorphism” hypothesis, which postulates that different host species act as different selective environments that maintain *ospC* diversity (14, 73). A critical study incorporating large-scale field-based genomic data robustly demonstrated a link between phylogenetic clusters of strains and particular host groups (13). This study sequenced the *ospC* locus of *B. burgdorferi* found in 533 *P. leucopus*, 92 passerine birds, and 628 individual nymphal ticks. They found strong patterns of host adaptation, revealing certain *ospC* genotypes were more likely to be found in rodents, some in birds, and others were generalists (Fig. 2). These findings have been partially experimentally reproduced, with results demonstrating that *ospC* type K strains (rodent associated) struggle to complete a robin-to-tick transmission cycle, while *ospC* type E strains (avian-associated) struggle to complete a *P. leucopus*-to-tick transmission cycle (69).

Notably, other work examined the frequency of *ospC* genotypes among hosts with greater granularity by examining frequencies of genotypes across different mammalian hosts (14, 59) and indeed found variation in *ospC* genotype frequency across mammals. We do note that other work has found that mammalian-species diversity alone likely does not play a major role in driving *B. burgdorferi* diversification. A study comparing a *Peromyscus*-dominated island to a species-rich mainland found lower *ospC* diversity on the island, but no increase in *Peromyscus*-adapted or human-invasive strains (74). These results suggest that while host-driven selection shapes *ospC* diversity, other forces, such as negative frequency-dependent selection (which predicts that low-abundance genotypes are selected for as populations become resistant to high-abundance genotypes [75, 76]) and the presence of dilution hosts, can impact *B. burgdorferi* population structure.

In summary, *B. burgdorferi*’s lack of control over its host destination is mitigated by a complex population structure, where different strains exhibit varying degrees of host flexibility, ensuring pathogen persistence across diverse and unpredictable ecological landscapes. An interesting consequence of more permissive hosts, such as *P. leucopus*, is that they not only amplify the pathogen’s prevalence but also promote pathogen diversity. As deermice are susceptible to infection by a wide array of *B. burgdorferi* strains (13), species-poor communities dominated by deermice are able to transmit a large variety of strains to ticks and thus enable the genetic diversity of the circulating spirochetes to be high (8). Among the strains that *P. leucopus* can transmit are the “generalist” RST1/OspC Type A/WGS 1 strains, which pose a significant human health risk (30, 36). Together, these findings highlight that what seems to be a single enzootic cycle is a complex web of intermingling, strain-specific subcycles driven by host adaptation.

### Studying diverse host-pathogen interactions in laboratory settings

While studies of *B. burgdorferi* in the wild can give key insight into how natural *B. burgdorferi* variation intersects with natural reservoir diversity, these studies are confounded by numerous variables, including co-infection with other pathogens, nutrition, age, and duration of infection. To better understand the impact of any single variable on enzootic cycling success, many researchers have successfully adapted laboratory tools to understand tick-to-reservoir-to-tick transmission. Primarily, this has included experimental studies of *Peromyscus* deermice (77–85) and/or traditional inbred *Mus musculus* laboratory models (83–87) (83–85) to understand how wild-type *B. burgdorferi* strains navigate the enzootic cycle. Sources of *P. leucopus* vary across laboratories, with some groups housing colonies derived from locally acquired deermice and others opting to standardize experiments across laboratories by using the “LL Stock”—a commercially available *P. leucopus* colony derived from 38 founders and maintained by the *Peromyscus* Stock Center at the University of South Carolina. Excitingly, recent work has unlocked CRISPR/Cas9 germline editing in *P. leucopus* by identifying a means to track the ovulation cycle of deermice (88, 89), which may lead to new mechanistic studies in how *P. leucopus* immunology interacts with *B. burgdorferi*. A smaller number of studies have attempted to understand *B. burgdorferi* enzootic cycling in avian models such as chickens (90), pheasants (91), quail (68), and robins (80).

Overall, enzootic cycling studies confirm that there is variability in how a given *B. burgdorferi* strain interacts with various hosts, as well as cross-strain differences within the same host. For instance, work by Zinck et al. demonstrated that across 11 strains of *B. burgdorferi*, there is a positive correlation between the ability for a given strain to colonize *M. musculus* (C3H/HeJ) tissues and its ability to be acquired and retained by new *I. scapularis* ticks (86). Rodent-to-tick transmission appeared to be sex biased, as more ticks acquired *B. burgdorferi* from male mice (86), which correlates with the higher bacterial burden observed in male mice in both this study and in subsequent work (92). Similarly, Lin et al. found that the initial and sustained burden of three *B. burgdorferi* strains in *P. leucopus* or robins partially predicted the ability for that strain to transmit to ticks (80). However, this relationship between bacterial burden and vertebrate-to-tick transmission was not observed in work examining transmission of a single strain of *B. burgdorferi* (strain B31, *ospC* Type A) in *P. leucopus* and *M. musculus* (83). We hypothesize that findings suggest that while the ability to reach a threshold bacterial burden is necessary for high vertebrate-to-tick transmission, bacterial burden alone is insufficient to describe variation in rodent-to-tick transmission. Evidence of this threshold may have been missed in the longitudinal study where only strain B31 was used, as the strain may remain above the required threshold for transmission while other factors work to suppress transmission. This contrasts with the Lin and Zinck studies, which compared multiple strains, some of which may have failed to reach the necessary threshold for transmission, demonstrating robust transmission phenotypes that mask the role of more subtle burden-independent phenotypes.

Critically, we and others have noted that there are differences in the capacity for *B. burgdorferi* to transmit from *Peromyscus species* to ticks and from inbred *M. musculus* to ticks (83–85), which is unsurprising given 25 million years of divergent evolution between these mice and deermice (93). An interesting finding is that *P. leucopus* is not universally better at enabling rodent-to-tick transmission of North American strains compared to inbred *M. musculus*, despite being a primary reservoir host—though, like many phenotypes discussed in this review, this phenomenon is strain specific. One explanation for these findings may be that because *P. leucopus* is continuously parasitized by *B. burgdorferi,* they have evolved fine-tuned mechanisms to contain the bacteria without clearing the infection entirely. While this specific hypothesis is speculative, these results firmly demonstrate that while *M. musculus* has served as a useful tool to study Lyme disease, their ability to model rodent-to-tick transmission in North America may be limited. Expanded use of the *P. leucopus* laboratory model is therefore necessary to better understand the selective pressures that North American *B. burgdorferi* strains face in nature.

Differences between *P. leucopus* and *M. musculus* immune responses to tick feedings have also been noted, with two primary observations. First, *P. leucopus* launches a more modest immune response against initial feeding larval ticks and better supports larval *I. scapularis* feeding than BALB/c mice (94). Second, *P. leucopus* develops enhanced immune activation during recurrent parasitization by larval and nymphal *I. scapularis* that disrupts larval *I. scapularis* (95), but not nymphal *I. scapularis* feeding (96), whereas such enhanced inflammation during recurrent feeding is not reported in *M. musculus* (97). Given that *I. scapularis* and *P. leucopus* have cohabitated the North American continent for millennia, it is perhaps not surprising that the *P. leucopus* immune response to *I. scapularis* differs substantially from the inbred *M. musculus* immune response. Fascinatingly, while the sensitized *P. leucopus* immune response against larval ticks is sufficient to disrupt overall feeding, it is insufficient to impact the acquisition of *B. burgdorferi*. The bacteria are not only able to survive this inflammatory milieu but also appear to thrive in it. Fisk et al. found that sensitized deermice exhibit higher rodent-to-tick transmission of *B. burgdorferi* than unsensitized rodents (95). What drives elevated rodent-to-tick transmission in these sensitized mice is unknown but warrants further examination. Separately, some work has sought to examine what factors lead to interspecific differences in initial larval tick adherence to hosts, with preliminary findings inconclusive on whether fur length affects tick adherence to a host, but a strong effect of the duration of contact and a moderate effect of speed on adherence was observed (98).

An interesting subset of laboratory studies have examined the dynamics of *B. burgdorferi* co-infection during the enzootic cycle. In *B. burgdorferi* endemic regions, reservoir hosts are exposed to numerous infected, feeding ticks both concurrently and recurrently. This can result in hosts and ticks that are coinfected with multiple *B. burgdorferi* strains (13, 47–51). Three distinct mechanisms can generate these coinfections. First, sequential infection occurs when a host is infected by one strain and then later exposed to a different strain via a subsequent tick bite. In laboratory experiments where *P. leucopus* deermice (78, 82) or *M. musculus* mice (87) are sequentially infected with two strains of *B. burgdorferi* weeks apart, rodent-to-tick transmission of the first strain does not change in response to the second strain. However, the ability of the second strain to establish infection and transmit is highly variable depending on the specific strains used. For some strain pairs, the first strain exhibits a strong “priority effect,” effectively blocking or suppressing the second strain. For other pairs, the outcome is more asymmetric, with one strain consistently outcompeting the other regardless of infection order.

Second, concurrent infection occurs when a host is fed upon by multiple infected nymphs at the same time or nymphs infected with multiple strains. Because both strains arrive together, neither has a priority advantage, and both can theoretically establish efficient infection. Curiously, while data largely do not support sequential infection being a major source of co-infected ticks, the low numbers of co-infected ticks that are generated could coinfect reservoirs through this mechanism, theoretically leading to future generations of coinfected ticks. However, this hypothesis needs to be tested. Interestingly, a recent study that co-infected *M. musculus* by simultaneously feeding ticks carrying different strains of *B. burgdorferi* did find that this method of co-infection did negatively impact the transfer of strain-specific antibodies from dams to pups (99). Third, co-feeding transmission occurs when multiple ticks feed on the same host at the same time. Ticks can acquire *B. burgdorferi* strains directly from co-feeding infected ticks without the host ever becoming systemically infected. This mechanism allows strain transmission independent of successful host colonization (51). How coinfection derived from these latter mechanisms impacts inter-*B. burgdorferi* competition and the overall reservoir competence of the host is not well understood.

Beyond examining enzootic cycling across hosts, there has been substantial interest in understanding how reservoir immune systems interact with *B. burgdorferi*. It has long been established from field (100, 101) and laboratory studies (77, 83, 102–104) that *P. leucopus* exhibits only very mild inflammation in response to *B. burgdorferi*, which does not affect their survival in the wild—with the exception of neonate deermice, which experience more severe symptoms following infection (103). This modest immune response by *P. leucopus* has also been quantified using RNA sequencing (83, 105, 106). While *Borreliaceae* do not have lipopolysaccharides (LPS) on their outer membrane, studies on *P. leucopus* immune responses to LPS demonstrate that *P. leucopus* also displays less inflammation in response to LPS than mice (107, 108) and rats (108). One posited explanation for the more moderate immune response by *P. leucopus* to a variety of stimuli is that the high-affinity immunoglobulin gamma Fc receptor 1 (CD64) is pseudogenized in *Peromyscus* species (109)*.* Beyond whole animal studies, recent additional work has sought to improve the ability to understand reservoir host biology *in vitro*, an important milestone for moving past the exclusive use of *M. musculus*. These studies have measured *P. leucopus* primary fibroblasts (80, 108, 110) and bone-marrow-derived macrophages (111) to understand responses to LPS and *B. burgdorferi*, as well as robin primary fibroblasts to study interactions with *B. burgdorferi* (80). Of note, Lin et al. infected *P. leucopus* and robin fibroblasts with multiple strains of *B. burgdorferi* and demonstrated that adhesion of different *B. burgdorferi* strains to either species’ fibroblasts predicted enzootic cycling success, but there was an inverse correlation between IFNγ and TNFα production by these cells and rodent-to-tick transmission (80).

Overall, these studies highlight the utility of using diverse hosts to better understand the *B. burgdorferi* enzootic cycle. The hurdles of integrating non-model systems into studying *B. burgdorferi* remain difficult, including adapting species to laboratory conditions and propagation, generating accurate genomes for molecular studies, and developing molecular tools (antibodies, cytokines, and cell lines) for mechanistic studies. Continuing to push past the technical hurdles of non-model reservoir hosts will likely provide key insights into how *B. burgdorferi* survives in nature, what selective pressures drive traits associated with pathogenesis, and how transmission in the wild and into humans can be disrupted. Already, *P. leucopus*-targeted vaccines (112–117), antibiotics (118, 119), and gene drives (88, 120) are being investigated to disrupt the enzootic cycle and prevent human spillover. Furthermore, there is interest in understanding how *P. leucopus* resists maladaptive inflammation in response to *B. burgdorferi* and LPS to potentially inform future treatments for humans to improve Lyme disease and sepsis outcomes.

## INTRASPECIFIC HOST DIVERSITY AND INTERACTIONS WITH *B. BURGDORFERI*

### Lyme disease in humans is a highly variable condition

Specific manifestations of Lyme disease vary immensely in humans, due to a combination of variables, including the infecting Lyme disease spirochete genospecies (121), but Lyme disease is most commonly characterized as causing a variety of broad symptoms, including fever, headache, and fatigue (122). Many acute cases of Lyme disease present with erythema migrans, a target-shaped red rash (“Bull’s-eye rash”), that spreads outward from the initial site of infection, which is frequently used as an identifier for infection (123). As the disease progresses, the bacteria spread via extremely brief forays into blood before exiting to various organs of the body, causing pain and weakness in muscles and more severe symptoms such as arthritis, meningitis, facial palsy, and, in rare cases, cardiovascular effects including atrioventricular blockage and cardiomyopathy (122, 124). The specific symptoms experienced appear to be sex biased, with men and women experiencing different symptoms, different severities of disease, and differences in diagnostic success (125–127). Treatment of Lyme disease most frequently consists of a course of oral doxycycline (128), which can also prevent Lyme disease altogether if used promptly following a tick bite (129). Antibiotic treatment resolves symptoms in most patients, though 5%–30% of patients experience antibiotic-refractory symptoms despite apparent successful clearance of the pathogen (128, 130–132). These long-lasting symptoms are classified as either antibiotic-refractory Lyme arthritis (133) or post-treatment Lyme disease syndrome (PTLDS) (134, 135), depending on whether joint or neurological symptoms are the predominant lingering symptoms. It is not yet fully understood what drives these differences in symptom resolution. Furthermore, it is important to note that not every human exposure to *B. burgdorferi* will result in a successful infection. As with natural reservoir diversity discussed above, some human-*B. burgdorferi* genetic combinations are recalcitrant to successful infection. Here, we explore attempts to understand the diversity of Lyme disease manifestations observed in both the development and resolution of symptoms before and following antibiotic treatment.

### Studying natural genetic diversity in Lyme disease-like illness across *Mus musculus*

Early studies on *B. burgdorferi* host-pathogen interactions used *M. musculus* inbred laboratory mice to explore how genetic factors may impact *B. burgdorferi*-induced disease. These studies made it immediately clear how differences in host genetics can drive variation in disease outcomes. C3H mouse strains were found to exhibit severe arthritic symptoms (136, 137), while BALB/cAnN only had swollen joints at high-dose bacterial inoculations (137). C57BL/6N had low-to-moderate inflammation independent of inoculating dose (136, 137). These differences in arthritic severity are mirrored in the development of carditis (136). Recent work has also further examined differences between these strains via RNA sequencing, finding more signatures of inflammation in C3H mice (138).

Using these cross-strain differences as a launching point, comparative biology approaches enabled researchers to identify IFNβ signaling as a primary driver of this accentuated pathology in C3H mouse strains, as upregulation of this cytokine leads to increased inflammation in joints (139). Forward genetic approaches were leveraged to identify *B. burgdorferi* arthritis-associated (Bbaa) loci in the genome, including Bbaa1. Work on Bbaa1 confirmed that when ARF, one of two proteins encoded by the gene *Cdkn2a*, was silenced using siRNA, *B. burgdorferi*-induced IFNβ was nearly completely ablated compared to wild-type animals (140). Subsequent research selectively blocked IFN cytokines using antibodies to further support the link between Type 1 IFN signaling and the development of arthritis (141). Other Bbaa loci were found to be independent of the IFNβ signaling pathway, as knockout of some did not impact IFN profiling, indicating that there are multiple pathways at play in promoting *B. burgdorferi* pathogenesis (139, 142). In addition to these studies, targeted genetic screens examined roles for the major histocompatibility complex (MHC) H-2 haplotype in driving *B. burgdorferi*-induced arthritis in mice and found the haplotypes H-2^k^ and H-2^d^ have no significant difference in the development of arthritis (143). This stands in contrast to human data, where MHC (HLA in humans) is thought to impact disease progression, as discussed below. This example is a reminder that, while mouse studies are often useful disease models, they are not always direct parallels for understanding Lyme disease in humans.

### Studying human genetic diversity during Lyme disease

**Fig 3:**
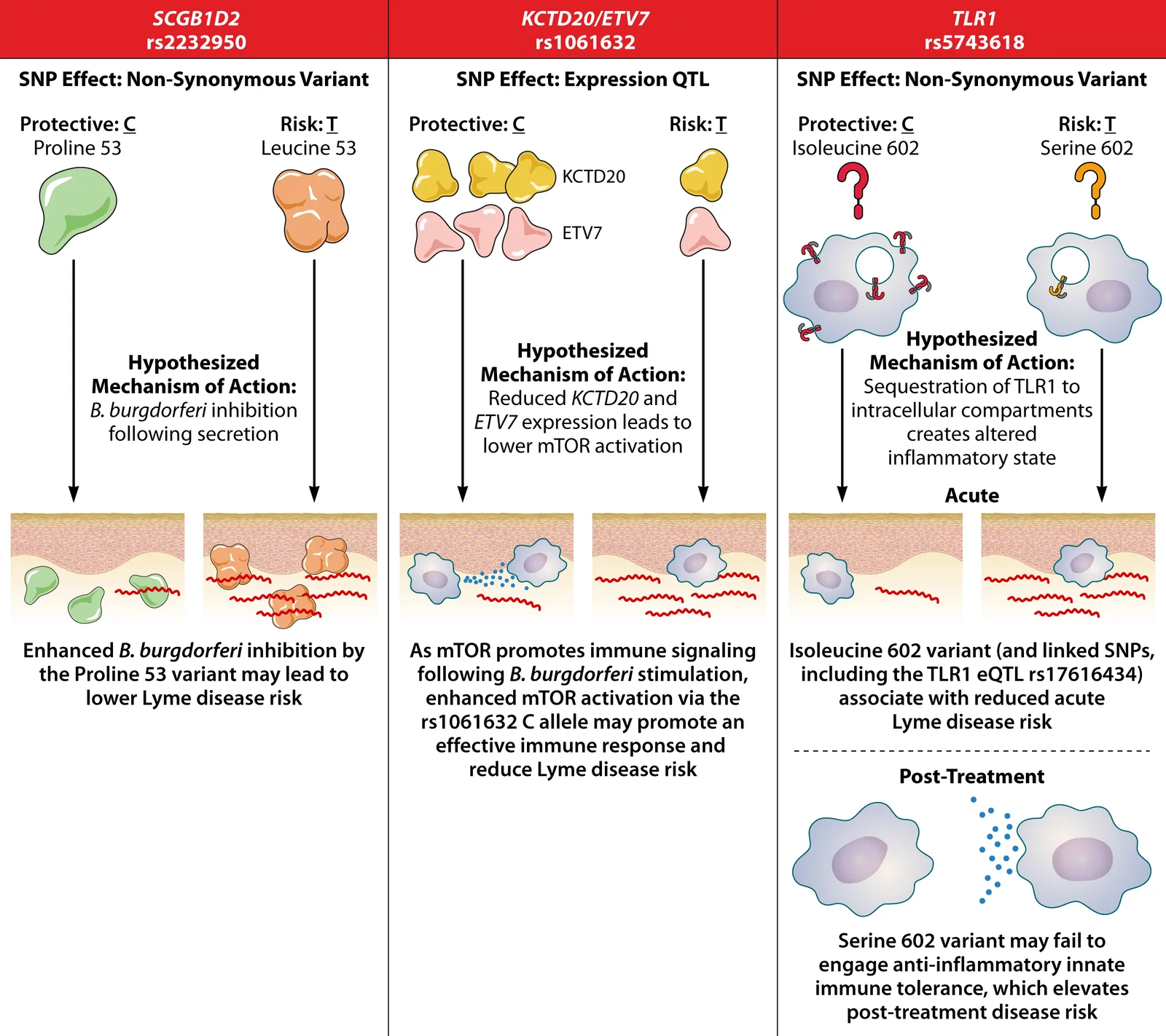
Human genetic diversity impacts Lyme disease risk and outcomes. Summary of three human single nucleotide polymorphisms (SNPs) that associate with Lyme disease and their proposed effects. Variation at rs2232950 causes an amino acid change in SCGB1D2, influencing the ability for the protein to kill *B. burgdorferi* and altering the risk of Lyme disease (148, 149). Variation at rs1061632 associates with variation in *KCTD20* and *ETV7* expression (expression quantitative trait locus [QTL]), which may impact mTOR activation and Lyme disease risk (149). Variation at rs5743618 results in an amino acid change in TLR1, which impacts TLR1 trafficking (150), detection of and response to *B. burgdorferi* (151–153), and risk of antibiotic-refractory Lyme arthritis (151, 152). Variation at this site also impacts the risk of acute Lyme disease, though it is unclear whether this variant is responsible or *TLR1* expression QTL (rs17616434) in high linkage disequilibrium with rs5743618 (148) drives this result. The figure is illustrated by Patrick Lane—ScEYEnce Studios.

Researchers have sought to understand how natural human genetic diversity impacts both susceptibility to infection and the manifestations of disease. Human genetic studies can be challenging as disease outcomes are dictated by complex interactions of host genetics, bacterial genetics, and environmental factors (144). Typically, this requires sampling thousands to hundreds of thousands of individuals to overcome confounding noise. Despite these challenges, recent studies have successfully deployed “The Awesome Power of Human Genetics” (145) to better understand Lyme disease susceptibility and severity. For instance, work with human cohorts has recapitulated findings from mice (146) in that patients with higher cholesterol and/or genetic risk of high cholesterol are at elevated risk of Lyme disease (147). Excitingly, hypothesis-free studies have also gained traction as Strausz and colleagues leveraged the FinnGen project and the Estonian Biobank to perform the first genome-wide association study (GWAS) identifying risk factors for Lyme disease (25,355 Lyme disease cases and 592,376 controls) (148). This study identified three loci that reached a genome-wide significant threshold (*P* < 5 × 10^−8^), indicating an association with Lyme disease risk, including rs2232950, a non-synonymous single nucleotide polymorphism (SNP) in *SCGB1D2* (Fig. 3). The authors demonstrated that SCGB1D2 is likely secreted in skin and inhibits *B. burgdorferi* growth. The risk allele at rs2232950 impairs this antibacterial function, leading to the hypothesis that this SNP contributes to variation in the skin’s ability to inhibit *B. burgdorferi* tick-to-human transmission. Additional genome-wide associations were observed in the *TLR1* locus (lead SNP rs17616434) and the *HLA* locus (lead SNP rs9276610). Shortly after the Strausz et al. study, a second GWAS by Vrijmoeth et al. utilized two cohorts (Discovery Cohort: 506 Lyme disease patients, 313 healthy controls, Replication Cohort: 557 Lyme disease patients, 441 controls) to identify genetic risk factors for Lyme disease (149). This group identified a single genome-wide association between rs1061632 and Lyme disease risk. The SNP was also associated with an increased pro-inflammatory cytokine response across all cohorts, which correlated with upregulation of both KCTD20 and ETV7 (149) (Fig. 3). As a result, the authors hypothesized that the upregulation of these genes reduces Lyme disease susceptibility, influencing Akt-mTOR-induced signal transduction and cytokine signaling. Furthermore, despite a considerably smaller sample size, this second GWAS noted a modest association between the *SCGB1D2* SNP (rs2232950, *P* = 4.4 × 10^−5^) and Lyme disease risk in their cohort.

While the studies above have focused on Lyme disease risk, additional work in human genetics has sought to understand risk factors for post-treatment Lyme disease sequelae. Due to difficulties in defining and recruiting large numbers of these patients, hypothesis-driven genetic studies on antibiotic-refractory Lyme arthritis patients make up much of the current work on post-treatment disease. These studies have noted associations between HLA DRB1*04 and DRB1*1501 alleles and susceptibility to antibiotic-refractory Lyme arthritis (154), as well as the *TLR1* non-synonymous SNP rs5743618 (151, 152) (Fig. 3). Notably, rs5743618 is in high linkage disequilibrium with the lead *TLR1* SNP (rs17616434) identified by Strausz et al. (148). Variation at rs5743618 results in either a serine or isoleucine at the final amino acid in the TLR1 cytosolic domain, with the serine variant preventing trafficking of the receptor to the plasma membrane (150). TLR1/2 heterodimers are pivotal sensors of *B. burgdorferi* infection (153, 155, 156), and cells from individuals with the serine-encoding variant display altered responses to *B. burgdorferi* (151, 152) (and other bacterial stimuli [150, 157, 158]) *ex vivo*, including a failure to deploy anti-inflammatory innate immune tolerance—a branch of innate immune memory. These data align with innate immune memory studies performed in mice, where an inability to deploy innate immune tolerance (159, 160) or a predisposition for pro-inflammatory innate immune training (161) is associated with more severe Lyme disease-like illness. Finally, despite the challenges associated with performing GWAS on these post-treatment disease cohorts, a recent study used electronic medical records paired with the MyCode genetic repository to conduct a GWAS on PTLDS patients (670 PTLDS cases, 2,653 controls) (162). This study identified two loci that reached a suggestive genome-wide statistical threshold (*P* < 5 × 10^7^): rs77857587 and rs10833979. While specific mechanistic links between these SNPs and PTLDS remain unclear, the study noted that the same SNPs did not associate with fibromyalgia, myalgic encephalomyelitis, or chronic fatigue syndrome, suggesting a unique etiology of PTLDS despite related symptomology with these other conditions.

Expanding the Lyme disease human genetic toolkit, Botey-Bataller et al. performed the first *ex vivo* human GWAS screen for cellular responses to *B. burgdorferi—*resulting in comparisons of PBMC and whole blood cytokine responses from 1,060 Lyme disease patients (153). Cytokine quantitative trait loci mapping was used to link distinct cytokine responses to genetic alleles. Of the 34 genome-wide significant SNPs identified, the group confirmed an association between the rs5743618 *TLR1* SNP and *B. burgdorferi*-induced cytokine expression (Fig. 3), as well as, curiously, the rs2232950 *SCGB1D2* SNP (Fig. 3). This latter association is hypothesized by the authors to be a consequence of higher bacterial burden in patients with the rs2232950 risk allele, in contrast to the more direct link between variation in *TLR1* and regulation of cytokine signaling. Overall, their study supports hypotheses that enhanced inflammatory signaling during early infection reduces Lyme disease risk. Comparable *ex vivo* approaches were used by Vrijmoeth et al. to enable targeted genetic testing between SNPs identified in their Lyme susceptibility cohort and cytokine responses (149). Together, these studies demonstrate the strength of using *ex vivo* tools to supplement human GWAS, as these combinatorial approaches can improve mechanistic understanding of *B. burgdorferi* infection phenotypes and disease outcomes.

Thus far, many human genetic studies on Lyme disease have focused on individuals of Western European descent, taking advantage of genetic homogeneity among this population to reduce statistical confounding variables. This practice results in blind spots for the effects of the disease among other ancestries, and thus, there is growing interest among researchers to expand efforts to include additional ancestries. To illustrate the importance of inclusive sampling, we note that the SNP most frequently referenced above (the TLR1 non-synonymous variant rs5743618 [148, 151–153]) has a high-risk allele frequency in European populations (0.71) but is considerably more rare in African (0.16), African American (0.17), Asian (0.05), East Asian (0.02), and Latin American (0.3–0.36) populations (163). Given the low frequency, it is very likely that other SNPs play larger roles in dictating Lyme disease risk and resolution among individuals outside of European descent, and future studies are therefore necessary to identify these genetic risk factors. This is not a critique of these past studies; rather, it is an acknowledgment that Lyme disease and post-treatment sequelae afflict all peoples living in endemic regions (162, 164), and future studies are needed to address current gaps in knowledge.

### Studying bacterial genetic diversity during Lyme disease

Following the identification of *B. burgdorferi* as the primary cause of Lyme disease in the United States (22), comparisons between disease in the United States and Europe, as well as the variation in symptoms driven by different *Borrelia* genospecies/*Borreliella* species in Europe, made it clear that Lyme disease spirochete diversity plays a major role in dictating disease outcomes (121). *B. burgdorferi* is primarily associated with arthritis (133, 165). *B. afzelii*, in contrast, is primarily associated with the skin rash acrodermatitis chronica atrophicans (166), common among late-stage European Lyme disease, and *B. garinii* with neuropathic symptoms such as meningitis (166). Some work suggests that variation in *ospC* across the genospecies may contribute to this variation (167). Within *B. burgdorferi* itself, there is considerable variation in whether a strain can cause disease. Using the genotyping methodologies described above (RST or *ospC* genotyping), researchers have identified associations between the ability for strains to disseminate and cause severe disease (30, 36, 152, 165) (Fig. 2). As a notable outlier, *ospC* type A (RST 1) strains cause the highest *in vitro* inflammatory cytokine response among *B. burgdorferi* strains (152). Similarly, they are responsible for the most severe symptoms among patients (30), are found in the blood at a higher density than other strains (165), have the highest capacity for dissemination (36), and have a higher likelihood of driving antibiotic-refractory Lyme arthritis (168), leading to the reputation of this lineage as the most virulent of the *B. burgdorferi* strains. This variation in disease severity is also seen in *M. musculus* mouse models infected with diverse strains, where, again, RST1 strains appear to be more invasive and pathogenic (169, 170). Unlike variation across genospecies, some data suggests intra-*B. burgdorferi* trophism is not determined by variation in *ospC* (171).

Recent work has also examined the impact of *Borrelial* co-infection on disease manifestations. Co-infection of mice with different Lyme disease spirochetes (*B. burgdorferi* and *B. garinii*) alters infection dynamics and disease severity (172). During co-infection, *B. burgdorferi* reached higher bacterial loads in mouse tissues and caused more severe arthritis and carditis relative to mice infected with *B. burgdorferi* alone or *B. garinii* alone. On the other hand, *B. garinii* loads were lower during co-infection than in single infections, which suggested that *B. burgdorferi* outperforms *B. garinii* in this mouse model. Studies aimed at understanding the dynamics and impacts of these co-infections during human infections are needed.

Of note, the disease state of a patient is a consequence of the *interaction* of host and bacterial genetics. This was beautifully demonstrated by Strle et al. and their study on natural genetic variation at the TLR1 SNP rs5743618. The authors demonstrated that the patients (i) with the serine-encoding risk variant and (ii) infected by an RST1 strain of the bacteria were at higher risk of severe inflammation, disease symptomology, and post-treatment disease (152). This was not true with other bacterial genotypes, demonstrating that host risk factors for post-treatment disease, in the absence of appropriate bacterial risk factors, are insufficient for antibiotic-refractory Lyme arthritis. As a result, knowledge of *B. burgdorferi* strain genetics alongside human data could provide consequential information in future human GWAS. Regardless, a common theme uniting many of the papers cited above is that we are currently in a very exciting period for understanding how natural genetic diversity impacts Lyme disease outcomes.

## CONCLUSIONS

In nature, the interaction between borrelial strain diversity and vertebrate host diversity creates a stable, resilient transmission system. Notably, while the classic dilution effect assumes that high vertebrate biodiversity reduces disease risk by adding incompetent hosts that divert tick meals away from competent hosts (7, 8), we now have an improved understanding of how this model is complicated by strain diversity. An incompetent host for one strain may be a competent reservoir for another (13). Therefore, host community composition can have a direct impact on human health risk by impacting the specific collection of *B. burgdorferi* strains present in a region. Given that many environments are home to numerous potential reservoirs, an ongoing question is what exact selective pressures may promote *B. burgdorferi* strain evolution toward generalization or specialization. The existence of both host-generalist and host-specialist strains poses the evolutionary question of whether broad flexibility, or the ability to survive in many hosts, is a “good idea.” Generalists may have more transmission opportunities, but specialists may achieve higher within-host fitness and more efficient reproduction in their preferred host. Furthermore, specialization may pose additional risks as different stages of ticks favor different hosts (173). Balancing these risks appears to maintain both strategies in the population.

Furthermore, while ecological studies have given insight into the links between *ospC* and host specificity (13), these studies leave considerable room for further exploration. For instance, given that *ospC* expression is dramatically reduced in *B. burgdorferi* during late-stage vertebrate infection (174), it is possible that OspC is not directly responsible for observations of low vertebrate-to-tick transmission of mismatched specialist strains during late infection (80). The dependence of *ospC* genotype on host specialization could be tested via knock-in experiments, in which a genotype of one strain is replaced either with a generalist or specialization-associated *ospC* genotype (e.g., swap *ospC* type E for *ospC* type K) and measuring the impact on the completion of the enzootic cycle in birds and rodents. As discussed above, beyond *ospC*, complement evasion remains a dominant hypothesis for describing host trophism (68, 80), but exploring other potential mechanisms remains an area of active research. Additionally, while many, but not all (14, 175), studies have focused on host adaptation to *Peromyscus* and avian hosts, advances in molecular biology have made it possible to more thoroughly ask whether certain *B. burgdorferi* genotypes associate with other species (e.g., shrews). Further attention should also be paid to how intraspecific variation within reservoir species impacts the *B. burgdorferi* enzootic cycle, as well as how different genetic makeups of ticks may play key roles in *B. burgdorferi* acquisition and transmission.

While the selective pressures *B. burgdorferi* faces during the enzootic cycle are, in themselves, fascinating, for many, the interest in understanding how *B. burgdorferi* survives is underscored by what these selective pressures promote following zoonotic spillover into humans. Humans are (typically) a dead-end host for *B. burgdorferi*—primarily because infected humans (i) receive antibiotics following infection, (ii) are rarely parasitized by large numbers of larval ticks, (iii) are likely to remove and kill ticks prior to their completion of feeding, and (iv) spend considerable time indoors, where humidity is far too low (<80% [176]) to support tick survival post-feeding, and (v) human-to-tick transmission appears to be exceedingly low (177). Therefore, the variation in host-pathogen interactions we observe as a result of pathogen genetics during Lyme disease (30, 36, 152, 165) cannot be the result of *B. burgdorferi* co-evolving with humans. Thus, reservoirs exist as incubators for human infectious disease, selecting for different genetic makeups of *B. burgdorferi*, which may, incidentally, cause more severe disease in Lyme disease patients. This leads us to hypothesize that understanding the selective pressures that influence the *B. burgdorferi* enzootic cycle could be useful in identifying bacterial processes that drive more severe disease.

In conclusion, we have sought to describe some of the intertwined layers of natural genetic diversity that impact *B. burgdorferi* survival and pathogenesis and highlight some areas of future research. We note that many of the new insights in understanding this variation have emerged only recently, thanks in large part to advances in DNA sequencing. Beyond new sequencing technologies, coordinated efforts between clinicians, geneticists, and immunologists have also helped accelerate studies aimed at understanding how human genomics impacts Lyme disease risk and severity, as has coordination between geneticists, microbiologists, and ecologists in improving our knowledge of natural transmission cycles. While Lyme disease was discovered in the 1970s (178), *B. burgdorferi* was isolated in the 1980s (22), and the initial studies of reservoir competence were completed by the early 2000s (8), there likely has never been a more exciting time to study how natural diversity affects *B. burgdorferi*-host interactions.
